# Socioeconomic and Geographic Patterning of Smoking Behaviour in Canada: A Cross-Sectional Multilevel Analysis

**DOI:** 10.1371/journal.pone.0057646

**Published:** 2013-02-28

**Authors:** Daniel J. Corsi, Scott A. Lear, Clara K. Chow, S. V. Subramanian, Michael H. Boyle, Koon K. Teo

**Affiliations:** 1 Harvard Center for Population and Development Studies, Harvard University, Cambridge, Massachusetts, United States of America; 2 Faculty of Health Sciences, Simon Fraser University, Vancouver, British Columbia, Canada; 3 The George Institute for Global Health, University of Sydney, Sydney, New South Wales, Australia; 4 Department of Society, Human Development and Health, Harvard School of Public Health, Boston, Massachusetts, United States of America; 5 Department of Psychiatry and Behavioural Neurosciences & Offord Centre for Child Studies, McMaster University and Hamilton Health Sciences, Hamilton, Ontario, Canada; 6 Population Health Research Institute, McMaster University and Hamilton Health Sciences, Hamilton, Ontario, Canada; CUNY, United States of America

## Abstract

**Objective:**

To describe the socioeconomic and geographic distribution of smoking behaviour in Canada among 19,383 individuals (51% women) aged 15–85 years.

**Methods:**

Current smoking and quitting were modeled using standard and multilevel logistic regression. Markers of socioeconomic status (SES) were education and occupation. Geography was defined by Canadian Provinces.

**Results:**

The adjusted prevalence of current smoking was 20.2% (95% confidence interval [CI]: 18.8–21.7) and 63.7% (95% CI: 61.1–66.3) of ever smokers had quit. Current smoking decreased and quitting increased with increasing SES. The adjusted prevalence of current smoking was 32.8% (95% CI: 28.4–37.5) among the least educated compared to 11.0% (95% CI: 8.9–13.4) for the highest educated. Among the least educated, 53.0% (95% CI: 46.8–59.2) had quit, rising to 68.7% (95% CI: 62.7–74.1) for the most educated. There was substantial variation in current smoking and quitting at the provincial level; current smoking varied from 17.9% in British Columbia to 26.1% in Nova Scotia, and quitting varied from 57.4% in Nova Scotia to 67.8% in Prince Edward Island. Nationally, increasing education and occupation level were inversely associated with current smoking (odds ratio [OR] 0.64, 95% CI: 0.60–0.68 for education; OR 0.82, 95% CI: 0.77–0.87 for occupation) and positively associated with quitting (OR 1.27, 95% CI: 1.16–1.40 for education; OR 1.20, 95% CI: 1.12–1.27 for occupation). These associations were consistent in direction across provinces although with some variability in magnitude.

**Conclusion:**

Our findings indicate that socioeconomic inequalities in smoking have persisted in Canada; current smoking was less likely and quitting was more likely among the better off groups and in certain provinces. Current prevention and cessation policies have not been successful in improving the situation for all areas and groups. Future efforts to reduce smoking uptake and increase cessation in Canada will need consideration of socioeconomic and geographic factors to be successful.

## Introduction

Smoking is the leading cause of death in high income countries such as Canada [1] and is a major risk factor for cardiovascular disease and cancer. [2], [3] In Canada, approximately 20% of all deaths are attributable smoking according to 2005 estimates. [4] The majority of these deaths are due to the following causes: lung cancer, chronic obstructive pulmonary disease and ischemic heart disease. [5] About 50% of smokers die of smoking-related diseases and smokers who die between the ages of 35 and 69 in Canada lose on average more than 20 years of life.[6]–[8] Importantly, however, smoking cessation can reverse the risk for mortality; quitting by age 50 can halve the lifetime risk, while quitting by age 30 can reduce the risk close to that of never smokers. [9], [10].

In 2010, the Canadian Tobacco Use Monitoring Survey (CTUMS) reported an overall smoking prevalence of 17% in the Canadian population (aged 15 years and older), down from 25% and the lowest since the surveys began in 1999 [11]; however the rate of decline appears to have slowed in recent years. [12] Although declines in the rates of smoking are good news, the overall trends may hide important socioeconomic and/or geographic variation. Uncovering such variation is key to informing tobacco control policies and identifying areas where more or differing strategies are required to increase smoking cessation and decrease smoking uptake.

Previous studies have indicated that the distribution of smoking is not uniform across the Canadian population. Geographically, rates of smoking vary considerably, with a higher prevalence of current smoking generally found in the Eastern and Atlantic provinces compared to Ontario and British Columbia. [12] In addition, smoking has consistently been found to be concentrated among individuals of lower socioeconomic status (SES) in Canada [13] and other high income countries[14]–[16]; while higher SES has been related to increased smoking cessation.[17]–[20] For example, evidence from the National Population Health Survey in Canada indicated that high levels of education and household income were associated with quitting over a two year period in men and women. [21] Despite these important findings, many questions remain including: to what extent are socioeconomic differences a source of variation in current smoking and quitting across provinces? And is the between-provincial variation consistent for all SES groups? Identifying geographic variation that is independent of individual characteristics and the consistency of this variation across SES groups will be an important step in tailoring future tobacco control priorities and/or priorities for resource allocations to programs aimed at tobacco use prevention and/or cessation. Further, it has not previously been shown whether the SES-smoking and SES-quitting relationships are qualitatively similar in both direction and magnitude across Canadian provinces. Identifying provinces where the gradients are shallower may be indicative of the success of certain programs aimed at tobacco use prevention and/or cessation in reaching all SES groups or suggestive of other social programs which aim to reduce overall inequalities in the provinces.

In this study, we examine the socioeconomic and geographic patterning of current smoking and quitting in Canada using the most recent and nationally representative survey on smoking. In addition, we assess the consistency of the SES-smoking and SES-quitting associations across Canadian provinces using education and occupation as markers of SES.

## Methods

### Data

The data are from the Canadian Tobacco Use Monitoring Survey (CTUMS), conducted in two cycles in the ten Canadian provinces from February to June and from July to December 2010. CTUMS was conducted by Statistics Canada on behalf of Health Canada to provide nationally representative data on tobacco use and related issues in Canada. [22] CTUMS covered all persons in Canada aged 15 and older except for residents of the Yukon, Northwest Territories, Nunavut, and those living in long-term care institutions or Canadian Forces bases. The sampling frame only included land line telephone numbers, thus excluding people without telephone land lines (about 16% of the target population). [22] The sampling weights provided with CTUMS have been adjusted to account for these individuals.

### Survey Design

A stratified two-stage sampling strategy was used in the CTUMS. [22] In each of the ten provinces, geographic strata were defined according to a census metropolitan area (CMA) stratum and a non-CMA stratum. CMAs are census defined areas corresponding to cities and urban areas with populations of 100,000 or more. In Prince Edward Island, only 1 geographic stratum was defined, and in Ontario and Quebec, a third stratum was defined for Toronto and Montreal, respectively. The CTUMS sampling frame was a list of in-service telephone prefixes (3 digit area code+next 5 digits) compiled from telephone company files within each of the province-stratum combinations. In the first stage, telephone prefixes were systematically sampled within each stratum and a random 2-digit number was appended to the prefix to form a complete telephone number. Known business and not-in-service telephone numbers were then screened and removed from the sample prior to dialing. In the second stage, and in order to increase the number of respondents in the 15 to 19 and 20 to 24 age groups, one or two individuals (or none) were selected to participate in the survey based on the age composition of the household. Sampling weights were provided with the CTUMS in order to adjust estimates for non-response, household composition, and an external adjustment to national population estimates from the Canadian census. [22] The household response rate (defined as the proportion of households who were reached and provided ages of all household members) was 73.8% for both cycles of the CTUMS from February to December 2010, and the individual response rate was 84.2%. [23].

Interviews for CTUMS were conducted using a computer-assisted telephone interviewing (CATI) application. The CATI application was employed in conjunction with extensive interviewer training in order to minimize data collection errors. [23] In total CTUMS collected information from 19,822 respondents aged 15–85 years in ten Canadian provinces. All respondents had complete information on current smoking status, age, gender, and province of residence. Respondents with incomplete information for any of the other independent variables (marital status, occupation, or education) were excluded (n = 439, 2.2%). An examination of the basic demographic characteristics between complete and partial respondents did not reveal any substantive differences. The final sample for analysis was 19,383.

### Outcome

Categories of smoking behaviour at the time of survey were defined as follows: current cigarette smokers were individuals who had smoked 100 or more cigarettes in their lifetime and reported smoking daily or occasionally during the past 30 days. Former smokers had smoked 100 cigarettes in their lifetime and reported having quit and did not smoke any cigarettes in the 30 days prior to the survey. Never smokers were lifelong never smokers (<100 cigarettes smoked in their lifetime). For these analyses, quitting was defined as the proportion of former smokers relative to ever smokers (current and former smokers). [24] Overall in the CTUMS sample, the weighted prevalence of current smoking was 16.57%; 26.6% were former smokers, and 56.8% were never smokers. Descriptive characteristics of the sample population by sex and categories of smoking behaviour have been tabulated in **Table 1**.

**Table 1:** Sample sizes and weighted estimates (%) of current smoking, former smoking, never smoking, and quitting for men and women across demographic and socioeconomic characteristics and province of residence.

	Men												Women						
Variables	Current smoker		Former smoker		Never smoker		Quit	Total	Current smoker			Former smoker			Never smoker		Quit	Total	
	n	%	n	%	n	%	%	n	n	%		n		%	n	%	%	n	
Total	1762	19.7	1886	29.3	5198	51.0	59.9	8846	1710	13.6		1990		24.0	6837	62.5	63.9	10537	
Age																			
15–19 yrs	375	13.1	51	2.0	2085	84.9	13.3	2511	284	10.6		39		1.4	2160	88.0	11.8	2483	
20–24 yrs	468	23.9	140	8.8	1176	67.4	26.9	1784	447	20.0		164		8.2	1377	71.8	29.0	1988	
25–44 yrs	388	24.5	275	18.2	803	57.3	42.6	1466	403	15.4		367		19.3	1169	65.3	55.5	1939	
45–64 yrs	439	20.3	833	38.3	833	41.4	65.4	2105	459	13.7		927		33.7	1280	52.6	71.1	2666	
65+ yrs	92	7.7	587	62.5	301	29.9	89.1	980	117	7.8		493		31.4	851	60.8	80.1	1461	
Marital status																			
Common-law/Married	625	17.3	1282	35.8	1498	46.9	67.4	3405	593	11.3		1150		27.8	2287	61.0	71.2	4030	
Widowed/Divorced/Separated	155	24.5	254	39.8	202	35.6	61.9	611	306	15.8		512		30.1	842	54.1	65.5	1660	
Single	982	23.8	350	12.2	3498	64.0	33.8	4830	811	17.8		328		11.3	3708	71.0	38.8	4847	
Education																			
Completed university	173	12.7	401	26.2	900	61.1	67.5	1474	174	7.3		426		20.2	1339	72.5	73.4	1939	
Completed college	358	18.1	385	25.9	1158	56.0	59.0	1901	447	13.5		571		27.1	1779	59.4	66.8	2797	
Completed secondary	700	25.5	588	32.5	1445	42.0	56.0	2733	615	17.4		673		27.3	1831	55.3	61.1	3119	
Less than secondary	531	23.1	512	33.7	1695	43.3	59.4	2738	474	17.0		320		18.1	1888	64.9	51.6	2682	
Occupation																			
Professional specialty	145	13.1	244	23.9	815	63.1	64.6	1204	236	8.4		367		22.7	1315	69.0	73.0	1918	
Not working	298	13.8	696	46.2	1151	40.0	77.0	2145	512	12.2		776		25.6	2054	62.3	67.8	3342	
Not reported	28	17.4	35	33.3	91	49.3	65.7	154	17	9.1		23		32.2	76	58.7	78.0	116	
Executive, managerial	168	17.9	218	28.8	565	53.3	61.7	951	297	15.6		409		27.5	1066	57.0	63.8	1772	
Sales or Service	388	20.8	214	17.1	1281	62.1	45.2	1883	566	19.6		352		18.2	2085	62.2	48.0	3003	
Manual	735	30.0	479	25.5	1295	44.5	45.9	2509	82	18.9		63		22.1	241	59.0	53.8	386	
Province																			
British Columbia	121	14.9	172	27.7	523	57.4	65.0	816	126	14.6		175		25.8	614	59.6	63.9	915	
Ontario	143	18.7	164	29.3	553	52.1	61.1	860	123	11.1		157		22.2	746	66.7	66.8	1026	
Prince Edward Island	153	18.8	226	35.0	476	46.2	65.1	855	135	13.0		223		27.3	685	59.7	67.8	1043	
Newfoundland	160	20.8	184	35.5	396	43.8	63.0	740	191	19.0		220		28.1	609	52.9	53.5	1020	
Quebec	189	21.0	211	32.5	499	46.5	60.7	899	162	13.7		203		27.4	679	59.0	66.7	1044	
Alberta	194	21.9	181	24.9	604	53.2	53.1	979	182	16.3		164		19.6	736	64.1	54.6	1082	
New Brunswick	178	22.0	180	33.3	444	44.8	60.2	802	170	15.6		199		25.2	640	59.2	61.8	1009	
Nova Scotia	193	22.7	208	30.9	531	46.4	57.7	932	194	18.8		196		22.9	673	58.3	55.0	1063	
Manitoba	219	24.2	171	23.8	640	52.0	62.0	1030	217	17.5		232		23.4	758	59.1	57.2	1207	
Saskatchewan	212	24.8	189	26.8	532	48.4	59.8	933	210	17.4		221		25.2	697	57.5	59.2	1128	

Canadian Tobacco Use Monitoring Survey 2010.

### Independent Variables

We considered age, sex, and marital status as demographic characteristics. Age was grouped into the following categories: 15–19, 20–24, 25–44, 45–64, 65+ years for descriptive analyses, and centred about its weighted mean (45 years) and treated as a continuous measure in regression models. In addition, polynomial terms were included for age to allow for the assessment of non-linearity. Sex was based on self-report. Marital status was categorized as common-law/married, single, or widowed/divorced/separated (reference: married). Socioeconomic status was measured by education and occupation. Education was grouped into four categories based on the highest level completed: less than secondary school, completed secondary, completed post-secondary/college, and completed university (reference: completed university). Occupational categories were adapted from the 2006 National Occupational Classification for Statistics (NOC-S) [25], and included professional specialties, executive or managerial positions, sales/service positions, and manual occupations (including trades, transport, industry, manufacturing, and utilities). Additional categories were specified for individuals not currently working and for respondents who did not report their occupation and professionals were taken as the reference category. Geographic location was defined as province of residence at the time of survey and verified by telephone company administrative files.

### Statistical Analysis

We used logistic regression to model current smoking (current smokers *vs* never smokers) and quitting (former smokers *vs* current smokers) conditional on age, sex, marital status, education, occupation, and province. Province of residence was ‘dummy’ coded and treated as a fixed classification in these models. To examine potential differences in smoking patterns between men and women, interaction effects were considered between sex and age, sex and education, and sex and occupation. Adjusted prevalence estimates were calculated for each independent variable separately while keeping the remaining independent variables at their mean values and expressed as a percent from 0.0 to 100.0. Next, models were extended by including a random effect for province and specifying two-level multilevel models.

The multilevel modeling strategy is described below, using the example of current smoking. Two-level models were specified with a binary response (*y*, current smoking *vs* never smoking) for individual *i* in province *j*. Current smoking Pr(*y_ij_* = 1), was assumed to be binomially distributed 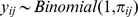 with probability 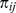 related to the set of independent variables 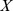 and a random effect for each level by a logit link function:
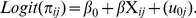
(1)


The right hand side of Equation 2 consists of the fixed part linear predictor (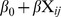) and random intercepts for provinces (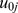). The intercept and the 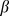-coefficients are interpreted as before in Equation 1. The set of independent variables remained consistent between models although the indicator variables for provinces were included in the random part of Equation 2 (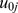). In this model, the random intercepts for provinces were assumed to be independently and identically distributed with variance 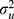. [26] The variance parameter quantifies heterogeneity in the log odds of smoking between provinces. We expressed the provincial-level variance as a percentage of the total variance from an initial model without covariates and from a final model accounting for all covariates.

In order to examine the consistency of provincial variation in current smoking and quitting by SES (defined by education and occupation) and to determine whether the SES-smoking and SES-quitting associations varied across provinces in terms of strength or direction, we expanded Equation 1 to allow the slope for SES to vary across provinces:
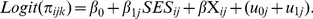
(2)


The key feature of Equation 2 is that the effect of education on smoking in province *j* consists of the overall average effect across all provinces (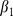), plus a province-specific (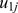) differential in this effect. We summarized this model by presenting the odds ratio for current smoking and quitting overall in Canada and for each province given a 1-category increase in education and occupation. The sampling weights provided with the CTUMS were used in all analyses. Logistic regression models were estimated with Stata (version 12.1) [27], [28] and multilevel models were estimated with MLwiN (version 2.26) using the second order penalized quasi likelihood (PQL) procedure. [29].

## Results

**Figure 1:**
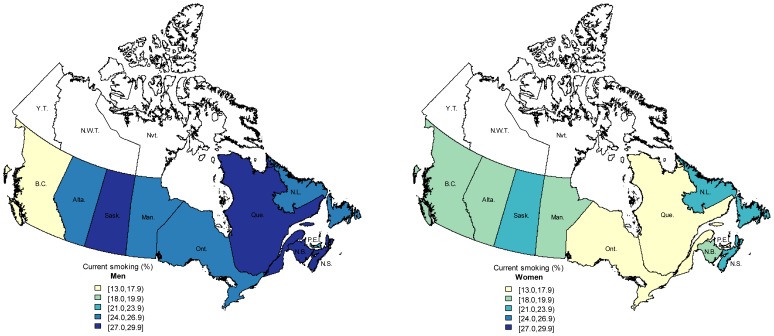
Adjusted prevalence of current smoking in Canadian provinces for men (left) and women (right) aged 15 years and above, Canadian Tobacco Use Monitoring Survey 2010. Darker colours indicate higher prevalence. Estimates adjusted for age, sex, marital status, occupation, education. Province name abbreviations: Alta. Alberta; B.C. British Columbia; Man. Manitoba; N.B. New Brunswick; N.L. Newfoundland; N.S. Nova Scotia; O.N. Ontario; P.E.I. Prince Edward Island; Que. Quebec; Sask. Saskatchewan; data not available for Yukon Territory (Y.T.), Northwest Territories (N.W.T), or Nunavut (Nvt.).

**Figure 2:**
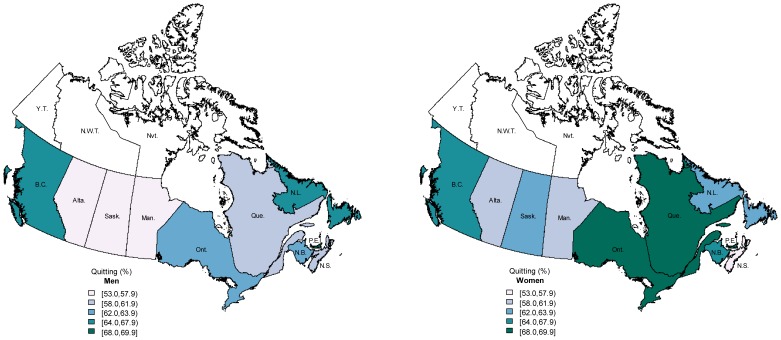
Adjusted prevalence of quitting in Canadian provinces for men (left) and women (right) aged 15 years and above, Canadian Tobacco Use Monitoring Survey 2010. Darker colours indicate higher prevalence. Estimates adjusted for age, sex, marital status, occupation, education. Province name abbreviations: Alta. Alberta; B.C. British Columbia; Man. Manitoba; N.B. New Brunswick; N.L. Newfoundland; N.S. Nova Scotia; O.N. Ontario; P.E.I. Prince Edward Island; Que. Quebec; Sask. Saskatchewan; data not available for Yukon Territory (Y.T.), Northwest Territories (N.W.T), or Nunavut (Nvt.).

In the 2010 CTUMS, the prevalence (adjusted for age, sex, marital status, occupation, education, and province) for current smoking among Canadians 15 years of age and older was 20.2% (95% confidence interval [CI]: 18.8–21.7) and 63.7% (95% CI: 61.1–66.3) for quitting. At the provincial level, current smoking varied from 17.9% in British Columbia to 26.1% in Nova Scotia (**Figure 1**), and quitting varied from 57.4% in Nova Scotia to 67.8% in Prince Edward Island (**Figure 2**). Odds ratios and 95% confidence intervals for current smoking and quitting from the mutually adjusted logistic regression models are presented in **Table 2**. The relationship between current regular smoking and age was strongly non-linear and this was emphasized by the statistical significance of the quadratic and cubic terms (P<0.001). This relationship had an inverse-U shape with a peak smoking prevalence found between the ages of 35 and 40 years. The prevalence increased rapidly at younger ages; it was 8.6% at age 15 and 27.8% at age 30, equivalent to a 3.3-fold increase (95% CI: 2.0–5.5).

**Table 2:** Mutually adjusted odds ratios and 95% confidence intervals from logistic regressions of current smoking and quitting on demographic and socioeconomic characteristics and province of residence.

	Current smoking			Quitting		
Variable	Odds ratio	95% CI		Odds ratio	95% CI	
Age						
10 year change	0.92	(0.82–1.04)		1.58	(1.37–1.82)	
Squared	0.82	(0.79–0.85)		1.04	(1.00–1.10)	
Sex						
Female	1.00			1.19	(0.95–1.49)	
Male	1.62	(1.35–1.94)		1.00		
Martial status						
Common-law/Married	1.00			2.01	(1.48–2.74)	
Widowed/Divorced/Separated	1.85	(1.42–2.43)		0.97	(0.67–1.41)	
Single	1.86	(1.44–2.41)		1.00		
Education						
Completed university	1.00			1.82	(1.24–2.68)	
Completed college	1.78	(1.34–2.36)		1.82	(1.28–2.58)	
Completed secondary	2.95	(2.23–3.91)		1.36	(1.00–1.86)	
Less than secondary	3.92	(2.78–5.52)		1.00		
Occupation						
Professional specialty	1.00			1.93	(1.30–2.87)	
Not reported	1.17	(0.57–2.42)		1.86	(0.89–3.90)	
Not working	1.39	(1.05–1.85)		1.12	(0.78–1.61)	
Executive, managerial	1.54	(1.14–2.10)		1.40	(0.94–2.08)	
Sales or Service	1.68	(1.25–2.25)		0.98	(0.67–1.45)	
Manual	2.05	(1.49–2.83)		1.00		
Province						
Ontario	1.00			1.43	(1.05–1.94)	
British Columbia	0.96	(0.74–1.26)		1.42	(1.05–1.92)	
Prince Edward Island	1.12	(0.87–1.45)		1.58	(1.20–2.09)	
Alberta	1.27	(0.99–1.62)		1.31	(1.00–1.71)	
Quebec	1.27	(0.99–1.64)		1.03	(0.78–1.35)	
Manitoba	1.32	(1.05–1.67)		1.05	(0.80–1.38)	
New Brunswick	1.35	(1.06–1.72)		1.37	(1.04–1.80)	
Newfoundland	1.42	(1.11–1.81)		1.08	(0.83–1.41)	
Saskatchewan	1.42	(1.12–1.80)		1.40	(1.07–1.84)	
Nova Scotia	1.58	(1.24–2.01)		1.00		

Canadian Tobacco Use Monitoring Survey 2010.

Men were more likely to smoke than women and had an odds ratio (OR) of 1.62 (95% CI: 1.35–1.94) for current smoking. In addition, those who were widowed, divorced, or separated (OR 1.85, 95% CI: 1.42–2.43) and singles (OR 1.86, 95% CI: 1.44–2.41) smoked more than married individuals. Quitters were more likely to be married (OR 2.01, 95% CI: 1.48–2.74) and women (OR 1.18; 95% CI: 0.95–1.49), although the OR for sex was not statistically significant at the conventional 5% level. Age was strongly associated with quitting; a 10 year change in age was associated with an increase of 1.58 in the odds of quitting and the non-linear terms were non-significant indicating that quitting generally increased with age in a linear fashion among surviving ever smokers.

### Socioeconomic Variation in Current Smoking and Quitting

**Figure 3:**
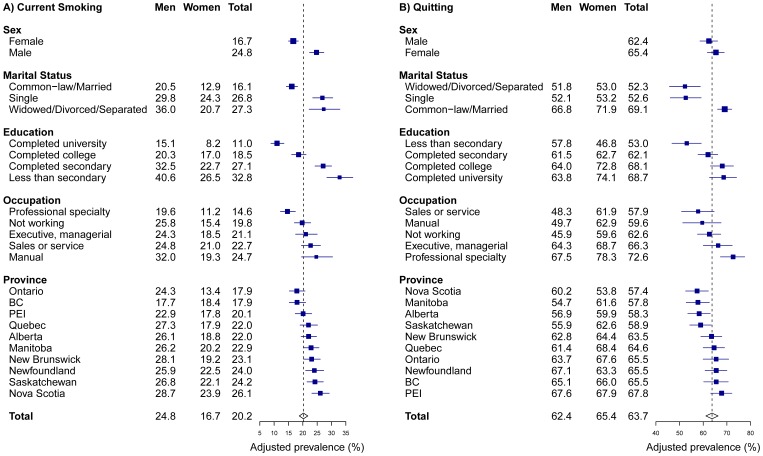
Adjusted prevalence of current smoking and quitting in Canada by demographic and socioeconomic characteristics and province. BC British Columbia; PEI Prince Edward Island.

A strong and graded association was observed between education and current smoking, with the odds of smoking being 3.92 (95% CI: 2.78–5.52) times higher among those who had not completed secondary school compared to those who had completed university (Table 2). There was no evidence of an interaction in this association by sex (P = 0.24). Current smoking was higher among those working manual occupations (OR 2.05; 95% CI: 1.49–2.83) and in sales or service occupations (OR 1.68; 95% CI: 1.25–2.25) compared to those in professional specialties with no indication of interaction by sex (P = 0.43). The adjusted prevalence of current smoking across all of the study variables and for men and women is presented in **Figure 3A**. We observed substantial variation in prevalence according to education; overall the prevalence varied from 11.0% among individuals who had completed university to 32.8% among those with less than high school education, corresponding to a difference of 21.8% (95% CI: 16.4–27.3). Large variation in the prevalence of current smoking was also observed by occupation group with a difference of 10.1% (95% CI: 4.2–16.3) between those in professional specialties (14.6%) and those in manual occupations (24.7%).

Similarly, we observed strong SES-quitting associations for education and occupation, with quitting being more likely among those with university education (OR 1.82) and in higher status occupations (OR 1.93 for professionals), although the overall educational gradient was not as pronounced as compared to current smoking (Table 2). In addition, there was indication of an interaction in the education-quitting association by sex, with the gradient being sharper for women compared to men (P = 0.02), although there was no indication of an interaction by sex in the occupation-quitting association (P = 0.20).

Conditional on all covariates, a 15.7% (95% CI: 6.7–24.3) difference was observed in the rate of quitting between those in highest and lowest educated groups overall, although this difference was 27.3% among women compared to 6.0% among men (**Figure 3B**). Overal a 14.7% difference was observed in the prevalence of quitting between those in professional (72.6%) and manual occupations (57.9%).

### Geographic Variation in Current Smoking and Quitting

A statistically significant difference in current smoking was observed between provinces in the logistic regression model treating provinces as a fixed effect (p = 0.0009). In this model, the odds of current smoking were greatest in Nova Scotia (OR 1.58; 95% CI: 1.24–2.01) and lowest in B.C. (OR 0.96; 95% CI: 0.74–1.26) compared to Ontario. Based on this model, the adjusted prevalence of current smoking varied from 17.9% in British Columbia and Ontario to 26.1% in Nova Scotia, equivalent to a difference of 8.2% (95% CI: 4.0–12.2). In addition, the prevalence of current smoking was lower in British Columbia and Ontario compared to the national average (Figure 3A). The adjusted prevalence of quitting across provinces was also calculated from a logistic regression model treating province as a fixed effect. From this model, a 10.4% (95% CI: 3.9–16.8) difference was observed in quit rates between the provinces with the highest rate (Prince Edward Island, 67.8%) and lowest rate (Nova Scotia, 57.4%). Nova Scotia, along with the western and prairie provinces (Manitoba, Alberta, and Saskatchewan) had quitter percentages lower than the Canadian average of 63.7%.

**Figure 4:**
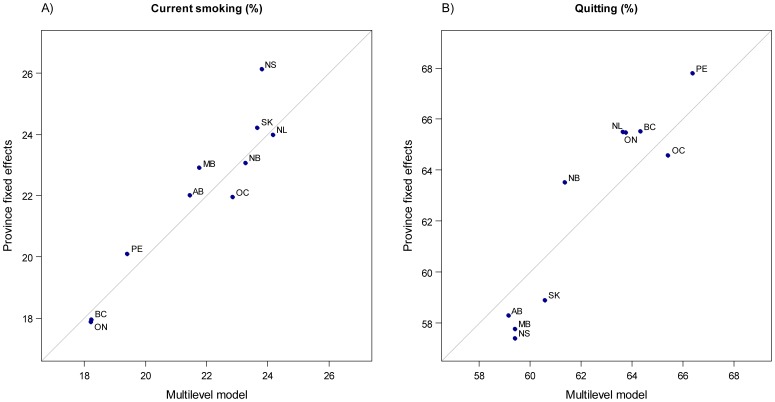
Comparison of adjusted prevalence estimates for current smoking and quitting for Canadian provinces based on mutually adjusted fixed effects and multilevel logistic regression models. Province name abbreviations: AB Alberta; BC British Columbia; MB Manitoba; NB New Brunswick; NL Newfoundland; NS Nova Scotia; ON Ontario; PE Prince Edward Island; QC Quebec; SK Saskatchewan.

We examined geographic variation in current smoking and quitting between provinces using a multilevel modeling approach. In this approach, provinces were treated as a random sample and between provincial differences in current smoking and quitting were assumed to come from a distribution estimated in the model. Compared to treating provinces as a fixed classification, the multilevel model yielded similar provincial-level estimates although the differences between provinces were found to be 2.2% narrower (6.0% *vs* 8.2%) for current smoking and 3.2% narrower (7.2% *vs* 10.4%) for quitting. The fixed effects estimates for each province, compared to the multilevel model estimates are shown for current smoking in **Figure 4A** and quitting in **Figure 4B**. The ordering of provinces was generally consistent in the two approaches. For current smoking, the provinces with lower than average rates of smoking (British Columbia, Ontario, and Prince Edward Island) in the fixed effects model also emerged as lower than average in the multilevel model, indicating the reliability of these estimates. The multilevel model tends to ‘shrink’ less reliable provincial estimates towards the national average; this is apparent in the quit rate model where a smaller range in the multilevel estimates for quitting was observed compared to the fixed effects approach (Figure 4B).

In addition to providing estimates of the between provincial differences in current smoking and quitting, the multilevel modeling approach allows for a more detailed examination of several research questions that are of substantive interest. These analyses revealed the amount of between-provincial variation in current smoking and quitting before and after accounting for individual characteristics (**Table 3**). In an initial random intercepts null model, provinces accounted for 0.9% and 1.1% of the total variation in current smoking and quitting, respectively. The addition of demographic and socioeconomic characteristics to the model reduced the variance in current smoking by 26.7% and in quitting by 25.7%.

**Table 3:** Variance in current smoking and quitting between provinces in Canada; expressed as percentage of the contribution to the total variance.

	Null model*			Fully adjusted model**		
Response	Variance	SE	%	Variance	SE	%
Current smoking	0.030	0.014	0.9	0.022	0.010	0.7
Quitting	0.035	0.016	1.1	0.026	0.005	0.8

Notes:

* Multilevel null model with random intercepts for province adjusted.

** Multilevel model with random intercepts for province and adjusted for age, sex, marital status, occupation, and education.

**Figure 5:**
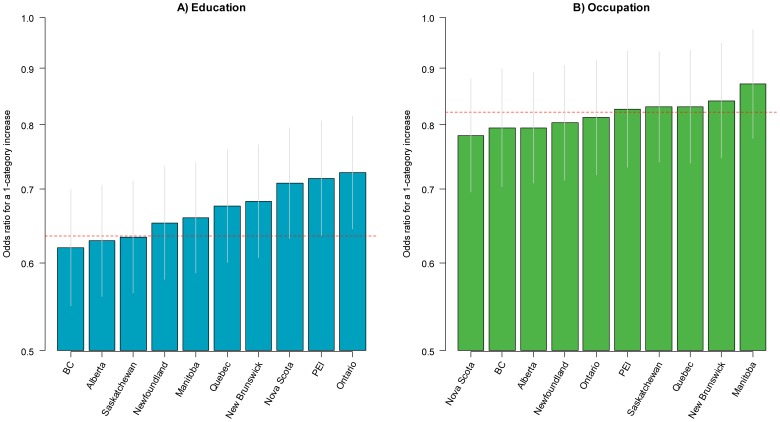
Odds ratios for current smoking for a one-category increase in the level of education and occupation across Canadian provinces. BC British Columbia; PEI Prince Edward Island.

**Figure 6:**
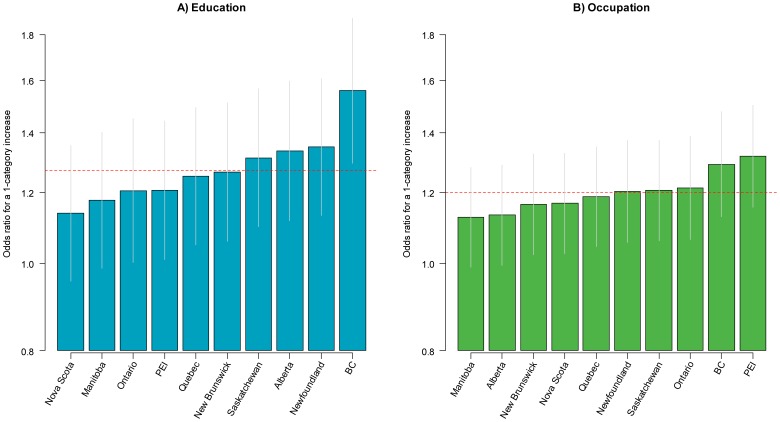
Odds ratios for quitting for a one-category increase in the level of education and occupation across Canadian provinces. BC British Columbia; PEI Prince Edward Island.

In order to assess consistency in the SES-current smoking and SES-quitting relationships across provinces, we estimated random-intercept, random slope multilevel models (Equation 2). In these models, the SES-current smoking and SES-quitting relationships were allowed to vary across provinces for education and occupation. The overall odds ratio for current smoking in Canada for a one-category increase in education was 0.64 (95% CI: 0.60–0.68) (**Figure 5A**) and 0.82 (95% CI: 0.77–0.87) for a one-category increase in occupation (**Figure 5B**). The direction of these relationships were consistent and statistically significant (p<0.05) in all provinces for both education and occupation. The magnitude of the association was greater than the national average in the provinces of British Columbia, Alberta, and Saskatchewan for the education relationship and in the provinces of Nova Scotia, British Columbia, Alberta, Newfoundland, and Ontario for the occupation relationship. In general, the magnitude of the SES-current smoking relationship was stronger for education compared to occupation. The associations between education and quitting and occupation and quitting were positive across all provinces, and statistically significant in 8/10 provinces for education (**Figure 6A**) and 9/10 provinces for occupation (**Figure 6B**). The overall odds ratio for quitting with each successive increase in the level of education was 1.27 (95% CI: 1.16–1.40) and 1.20 (95% CI: 1.12–1.27) for each successive increase in level of occupation. The education-quitting relationship was stronger than the national average in Saskatchewan, Alberta, Newfoundland, and was highest in British Columbia (OR 1.56, 95% CI: 1.29–1.88). The occupation-quitting relationship was stronger than the national average in Ontario, British Columbia, and Prince Edward Island. Associations were shallower than the national average in Quebec, Nova Scotia, Manitoba, and New Brunswick for both education and occupation; in Ontario and Prince Edward Island for education; and in Alberta for occupation.

## Discussion

This paper has four principal findings. First, current smoking in Canada was strongly influenced by socioeconomic status; people who had not completed secondary level education were more than three times as likely to smoke compared to those who had completed university. Second, geographic analyses revealed that the adjusted prevalence of current smoking was statistically significantly lower than the Canadian average in three provinces: British Columbia, Ontario, and to a lesser extent, Prince Edward Island. This finding was consistent when provinces were treated as a fixed classification and as a random classification in a multilevel model. In addition, the relationships between education and current smoking and between occupation and current smoking were consistent, negative, and statistically significant across all provinces in Canada. Third, although roughly six out of ten Canadians who had ever smoked had quit, quitting was more likely to occur among those of higher socioeconomic status. Geographically, large differences in quit rates were found between provinces, although the magnitude of difference was attenuated when province was treated as a random classification using a multilevel model. Forth, although the associations between education and quitting and between occupation and quitting were positive in all provinces, some heterogeneity in the magnitude was found, especially in the education-quitting relationship which was noticeably steeper in British Columbia and shallower in Nova Scotia compared to the national average.

There are some limitations in this work. First, the CTUMS data are cross-sectional therefore causal inferences from our findings must be interpreted cautiously. The primary motivation for this study, however, was to investigate variability in smoking behaviour across socioeconomic and geographic dimensions and such a design is appropriate. Second, the data are from a telephone survey, which as a design has several inherent potential sources of bias in terms of population coverage. For example, one limitation of telephone sampling is that some individuals either do not have telephones or have only a mobile phone, which were not part of the random telephone prefix sampling frames in the CTUMS. Estimates from the 2010 Residential Telephone Service Survey suggest that 14–16% of the Canadian population do not have a landline and that these individuals are concentrated among those of 18–34 years of age and with below-average income. [30] Although efforts were made to weight the CTUMS survey data for individuals without land lines, it is conceivable that some of the prevalence estimates may be biased downwards given that our findings suggest smoking to be higher among these ages and lower SES groups. In addition, telephone surveys can produce a potential reporting bias among younger respondents who may be prone to give socially desirable answers about their smoking habits in the presence of their parents or family. Estimates of youth smoking among 15–19 year olds in the CTUMS were lower than what has been reported among 16–19 year olds the UK (12% *vs* 24%) [31], although the CTUMS data for youth smoking have demonstrated good concordance with other prevalence estimates in Canada from general health surveys such as the Canadian Community Heath Survey, which uses a combination of in-person and telephone-based interviews.[32]–[34] Second, we only considered cigarette smoking in the present study. Socioeconomic and geographic differences for the use of cigars, or smokeless (chewing) tobacco were not considered these analyses although these forms of tobacco use may be important to consider among certain population groups in Canada. These forms of tobacco are, however, used less frequently and only in a minority of the Canadian population. [12] Further research on the patterning of occasional smoking in Canada, the use of other forms of tobacco, and potentially related factors such as alcohol use is needed.

The overall relationship observed between socioeconomic status markers and smoking in this study was similar to what has been reported previously in Canada. [13], [35], [36] We noted strong gradients in current smoking by level of education and occupation, which were minimally changed after adjustment for potentially confounding variables. Differences remained in rates of current smoking between provinces after accounting for demographic and socioeconomic characteristics in both the fixed and random effects models, although the estimated prevalence for several provinces (for example Nova Scotia and Manitoba) were ‘shrunk’ towards the national mean in the multilevel model. Due to the treatment of higher level units as part of a distribution, the multilevel approach is typically more conservative in estimating between group-differences. [26] The between provincial differences in quit rates were approximately a third narrower in the multilevel modeling approach, and the most obvious pattern of attenuation compared to the fixed effects model was found for provinces with quit rates lower than the national average. In this way, the random effects approach is favoured because it protects against the over interpretation of extreme group-level differences which are potentially less reliable.

Conditional on socioeconomic and demographic characteristics, province of residence was associated with <1% of the total variability in current smoking and quitting in the fully adjusted multilevel models. Although the magnitude of this variability was not large, adjustment for individual characteristics explained about one quarter of the provincial-level variation in current smoking and quitting, indirectly suggesting the potential relevance of geographic context in influencing smoking behaviour in Canada. [37], [38] Province was the only higher-level geographic unit that was available in the CTUMS; thus potentially important geographic variability in smoking behaviour at lower levels of aggregation (for example health regions, or communities) may have been masked in these analyses. [39].

Our study documents that current smoking in Canada follows an inverse gradient by SES which was consistent in direction across all provinces. Similarly, a consistent and positive gradient was observed with quitting for increasing SES. Interestingly, there was some variability in the magnitude of these associations, with larger variability observed for quitting. The education and occupation gradients appear to be stronger in British Columbia, a province with the lowest prevalence of current smoking and second highest quit rate. In comparison, the education gradients were considerably shallower in Manitoba, New Brunswick, and Ontario. This may indicate that programs aimed at tobacco use prevention and/or cessation or other social assistance programs have been better able to reach all SES groups in certain provinces compared to others. Further comparative analyses of provincial policies are required to understand why SES gradients vary across provinces.

Our findings related to the socioeconomic differentials in quitting are of public health importance. On average, individuals who where married, highly educated, and working in higher status occupations had the highest likelihood of quitting. While a positive SES-quitting relationship has been previously reported [17]–[20], the implications of these findings have been given less attention in recent years. Indeed, it has been suggested that policies aimed at reducing tobacco consumption may be responsible for widening the socioeconomic differentials in smoking, at least in the short-term. [15] Individuals with greater education and/or material resources may be more responsive to accessing health services in general [40] and this may extend to primary care and other sources of cessation support including telephone quitlines [41], medication, nicotine replacement, or counselling.

Interventions carried out at a population level including taxation, dissemination of health information and pictorial warnings on tobacco products, restrictions on use, advertisements, and sale of cigarettes have been effective at reducing average consumption [42], [43], although it is less clear whether these interventions are reaching all segments of the population. Indeed, there is evidence that taxation policies are being circumvented among some population groups and in some geographic areas. For example, a quarter of respondents in the Ontario Tobacco Survey reported recent purchasing of contraband cigarettes from First Nations reserves without paying applicable federal or provincial taxes. [44] In addition, the usual purchasing of contraband or low-tax cigarettes was more common among lower educated groups, heavy smokers, and those who do not intend to quit. [44].

In Canada, all provinces and territories have legislation restricting smoking in workplaces and public places including restaurants, bars, and public transportation. [45] Although such contextual factors were not explicitly considered in the present study, evidence from New Zealand suggests that such workplace restrictions may have been more effective in reducing rates of smoking and exposure to environmental tobacco smoke among those in professional occupations. [46] In addition, previous research in Canada has revealed the importance of social factors such as family norms discouraging smoking in explaining between-area differences in prevalence. [39] Successful efforts to increase smoking prevention and cessation across the entire Canadian population will therefore need explicit consideration of lower socioeconomic, Aboriginal, other disadvantaged groups along with contextual factors at the local and provincial levels. Policies such as tax increases and smoking restrictions may not be effective in increasing cessation among the poor or less educated without additional support or assistance in reducing tobacco dependence in these groups. In addition, further research is needed to understand the underlying causes of geographic variability in smoking behaviour in Canada. Such variation may be a result of different legislation or taxation but may also be influenced by different social or cultural norms across provinces. [47].

The persistence of high rates of current smoking and low quit rates in certain geographical areas and among certain socioeconomic groups in Canada indicates the failure of current smoking cessation policies to be effective in improving the situation for these areas and groups. Identifying these areas and groups is one step to examining the barriers to decreasing smoking in the population; further study is required to identify what barriers exist in these areas and what interventions may improve the situation.
